# Validation of the Effectiveness of Visual Feedback Systems in Respiratory Gating Carbon Ion Radiation Therapy

**DOI:** 10.1016/j.ijpt.2026.101319

**Published:** 2026-05-08

**Authors:** Ren Umetani, Yuya Miyasaka, Hikaru Souda, Hongbo Chai, Miyu Ishizawa, Yasuhito Hagiwara, Takashi Kaneko, Yoshifumi Yamazawa, Hiraku Sato, Masashi Koto, Takeo Iwai

**Affiliations:** 1 Department of Heavy Particle Medical Science, Yamagata University Graduate School of Medical Science, Yamagata, Japan; 2 Department of Radiology, Tsuchiura Kyodo General Hospital, Ibaraki, Japan; 3 Department of Radiation Oncology, Yamagata University Faculty of Medicine, Yamagata, Japan; 4 Department of Radiation Oncology, Yamagata University Hospital, Yamagata, Japan

**Keywords:** Carbon-ion therapy, Visual feedback, Respiratory motion, Respiratory-gated radiotherapy

## Abstract

**Background:**

This study aimed to evaluate the efficacy of respiratory-gated irradiation using visual feedback (VF) in carbon-ion radiotherapy (CIRT). Furthermore, in patients with liver cancer undergoing CIRT, the treatment times of treatments with and without VF (non-VF) were compared.

**Material and methods:**

Thirty-three patients with liver cancer were analyzed. VF system was applied in 10 patients with liver tumors. To evaluate the efficacy of VF, patients treated non-VF were selected as a control group. All treatments were performed under free-breathing conditions in both the VF and non-VF groups. For each VF patient, 1-3 candidate non-VF patients were identified by matching the CTV size and irradiation gate width (amplitude). From these candidates, 1 non-VF patient was selected as a matched control for each VF patient. The average treatment time for each VF patient was compared with that of the corresponding non-VF patient. The effect of the reduced treatment time for VF was calculated by comparing each VF patient with the corresponding non-VF patient. To quantify respiratory variability, the root mean square error and standard deviation were calculated.

**Results:**

The treatment time was reduced in 8 of 10 pairs. Further, in 6 of 8 pairs, the treatment time was reduced by more than 5 minutes when using VF compared with non-VF. The RMSE value was significantly reduced at 50% and 100% (*P* < .05). The RMSE value decreased by 28.2% in the VF compared with non-VF. VF was effective in improving respiratory waveform reproducibility in most pairs.

**Conclusion:**

The results suggest that VF contributes to a reduction in treatment time, indicating improved time efficiency during irradiation sessions. In addition, VF improves respiratory waveform reproducibility, which may contribute to enhanced treatment accuracy. Furthermore, shortening the treatment time may reduce the treatment burden on patients and improve overall treatment workflow.

## Background

Carbon ion radiation therapy (CIRT) is distinguished by its precise dose distribution, enabled by the Bragg peak, and its enhanced relative biological effectiveness, resulting from its high linear energy transfer. CIRT is used for treating refractory cancers that are resistant to conventional X-ray therapy and chemotherapy.^1^ The application of CIRT has been expanding to different types of cancers that involve organs such as the lung, liver, pancreas, and kidney, which move due to respiratory motion.^2^, ^3^, ^4^, ^5^, ^6^, ^7^, ^8^, ^9^­

Managing respiratory motion is a major challenge in radiation therapy for tumors in organs affected by respiration. Respiratory motion may cause underdosage to tumors and overdosage to normal tissues.^1^ The representative countermeasures for respiratory motion include breath-holding techniques, respiratory-gated irradiation, and tumor-tracking irradiation. In scanning particle therapy, gating irradiation with fast rescan is occasionally applied to reduce the interplay effect between tumor motion and particle scanning.^10^, ^11^, ^12^ In several cases of gating, the position of the abdominal surface is tracked as a sign of respiratory motion for gating, and its variation is shown as a respiratory waveform. The respiratory waveform changes significantly over time.^13^ During irradiation, the radiation therapist continuously monitors the patient’s respiratory waveform and adjusts the respiratory gating level to compensate for baseline shifts in breathing. During these adjustments, irradiation must be temporarily interrupted, which results in prolonged treatment time.^14^, ^15^ Prolonged treatment time may cause additional respiratory instability. The treatment times tend to be longer, particularly in patients with larger tumors. A shorter treatment time achieved by stabilizing the gating position of the respiratory motion is beneficial for both patients and hospitals. Visual feedback (VF) is an effective method for maintaining a stable respiratory pattern.^16^, ^17^, ^18^, ^19^, ^20^, ^21^ Hence, this study used VF to achieve respiratory stability.

VF involves the patient controlling his/her breathing while visually monitoring their own respiratory waveform. Various VF systems for X-ray radiation therapy have been developed.^16^ Nakajima et al. showed that various VF systems used in X-ray radiation therapy can improve respiratory stability and reproducibility compared with free breathing without VF.^16^ Regarding treatment time, Linthout et al revealed that applying VF significantly enhances the efficiency of respiratory-gated radiotherapy.^17^ Further, other studies have found that VF is effective in reducing variability in respiratory amplitude, suppressing baseline shifts, and decreasing the frequency of respiratory adjustments during treatment.^18^, ^19^, ^20^, ^21^, ^22^, ^23^, ^24^, ^25^ However, these studies have focused on X-ray radiation therapy. In CIRT, synchrotrons are currently the only accelerator type available for clinical use.^26^ Therefore, because beam delivery in synchrotrons is inherently intermittent, respiratory irregularities decrease the likelihood that beam availability coincides with the gating window, leading to prolonged treatment time.^27^ Even when patients breathe similarly to those receiving photon therapy, the physical characteristics of synchrotron-based delivery may result in longer treatment times.^27^, ^28^ Hence, it is essential to improve the reproducibility of the respiratory amplitude to ensure time efficiency in irradiation sessions. However, no studies have evaluated the effects of VF on respiratory reproducibility and reduced treatment time in actual patients receiving CIRT.

The current study aimed to develop a VF system for CIRT and validate its efficacy in improving respiratory reproducibility and reducing treatment time. This study focused on patients with hepatic tumors who are suitable candidates for CIRT. However, hepatic tumors are highly susceptible to respiratory-induced motion due to their proximity to the diaphragm. Therefore, this population provides an appropriate model for evaluating the applicability of the VF system under conditions with pronounced respiratory motion.

## Materials and methods

### Patient selection

The institutional review board (2023-113) approved this study. From April 1, 2024, to October 31, 2024, our VF system was applied in the treatment of 10 patients with liver cancer or metastatic liver tumors at our hospital. Written informed consent was obtained from all patients prior to treatment. The 3 exclusion criteria for patient selection were as follows: 1) inability to understand the treatment, 2) treatment in the prone position, and 3) inability to visually recognize the monitor.

To evaluate the efficacy of VF, non-VF cases were retrospectively selected as controls from a historical cohort of patients treated without VF at our institution. Non-VF patients were those who received CIRT at our hospital between October 2022 and June 2024. For each VF patient, 1-3 non-VF patients were selected by matching the CTV size and irradiation gate width (amplitude) to each corresponding VF patient. The non-VF cohort consisted of 17 patients with hepatocellular carcinoma, 3 with intrahepatic cholangiocarcinoma, and 2 with metastatic liver tumors. The age ranged from 48 to 90 years (mean, 77 years), including 20 males and 3 females

The VF cohort consisted of 9 patients with hepatocellular carcinoma and 1 with intrahepatic cholangiocarcinoma. The age ranged from 67 to 92 years (mean, 77 years), with 7 males and 3 females.

### Development of visual feedback system for carbon-ion radiotherapy

**Figure 1:**
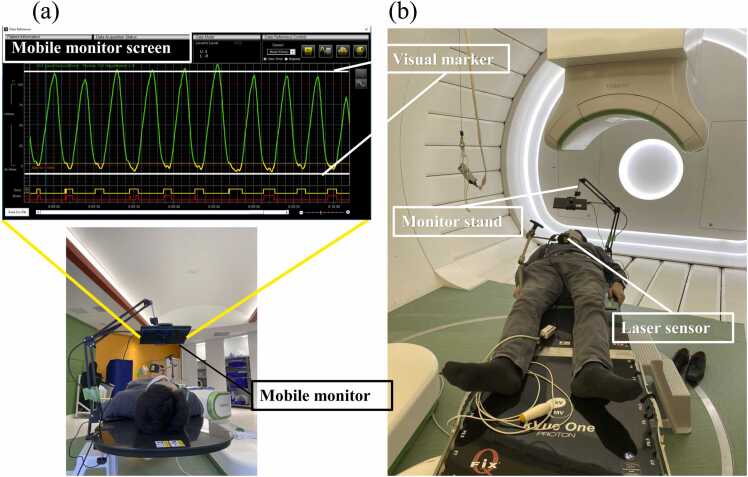
The VF system displays the respiratory waveform by tracking abdominal motion induced by respiration using an infrared laser sensor.

Figure 1 shows the newly developed VF system. The patient’s respiratory waveform was obtained by detecting the abdominal wall motion using a respiratory gating system (AZ-733VI, Anzai Medical Co, Ltd) with a laser tracking sensor option. As shown in Figure 1, the respiratory waveform was displayed on a monitor during the entire irradiation session. The patients performed breathing exercises while following the visual markers displayed on the monitor. The monitor device was set up within 1 minute prior to treatment. Patients received approximately 5 minutes of breathing training during the planning CT session. During treatment, the monitor displayed visual markers indicating the predefined target ranges of inhalation and exhalation, and patients were instructed to maintain their breathing within these target ranges. The time delay between motion detection by the laser sensor and display on the monitor was less than 100 ms. The monitoring system was designed to maintain respiratory amplitude reproducibility within the predefined gating window (<5 mm), which was consistent with the uncertainty margins used for treatment planning.

### Treatment planning

Patients were immobilized using a thermoplastic sheet (MTPLHA01, CIVCO) and a vacuum cushion (P10104-129, Elekta) in the supine position with arms raised. Four-dimensional computed tomography (4DCT) was performed using Aquilion ONE (Canon Medical Systems, Otawara, Japan) with 10 respiratory phases, where 0% and 90% corresponded to end-inspiration and 50% corresponded to end-expiration. Treatment planning was conducted using RayStation 10A (RaySearch Laboratories, Stockholm, Sweden). Gross tumor volume (GTV) was delineated by experienced radiation oncologists, and a margin of 5-7 mm was added to define the clinical target volume (CTV).

Tumor motion was evaluated across the respiratory phases of the 4DCT by analyzing the center-of-mass displacement of the CTV. Based on this analysis, a patient-specific respiratory-gating amplitude was determined to limit tumor displacement to within 5 mm. CIRT was delivered using raster scanning with a full-energy scan.^28^ The prescribed dose was 60 Gy (relative biological effectiveness) in 4 fractions.^29^, ^30^, ^31^ Robust optimization based on our institutional protocol with a setup uncertainty of 2 mm and a range uncertainty of 2% was applied.^32^, ^33^, ^34^ In CIRT, patient positioning is performed using immobilization devices and bone matching with orthogonal X-ray imaging, and previous studies have reported that the residual setup error with these procedures is approximately 1-2 mm.^34^ Based on this level of positioning uncertainty, a setup uncertainty of 2 mm was applied in the present study. Dose calculation was performed using a pencil beam algorithm with a modified microdosimetric kinetic model.^35^

### Clinical workflow

In both the VF and non-VF workflows, continuous respiratory monitoring and the possibility of beam interruption were required to ensure patient safety. Target reproducibility was clinically ensured using respiratory gating, continuous monitoring, and image-guided positioning with implanted fiducial markers. For image-guided positioning, X-ray images were acquired before each irradiation session and matched with digitally reconstructed radiographs to verify target position. Respiratory-gated irradiation was also applied in the non-VF (historical patient) cohort using the same laser-based gating system, and the only difference between the workflows was that VF was implemented during CT simulation and treatment delivery in the VF cohort. If marked respiratory irregularity occurred, beam delivery was interrupted by the radiation therapist in both groups.

The primary difference between the workflows was the use of the VF system during CT simulation and treatment delivery. In the VF workflow, the VF system was applied during the planning CT simulation session, and treatment plans were generated based on respiratory waveforms stabilized with VF. In both the VF and non-VF workflows, treatment planning was performed by medical physicists.

During treatment delivery, patient setup, respiratory monitoring, and beam control were performed by radiation therapists in both workflows. Although the VF workflow required placement of the VF monitor during setup, the installation was simple and required less than 1 minute. Therefore, the overall setup time was comparable between the VF and non-VF workflows.

In the non-VF workflow, respiratory monitoring was performed by tracking abdominal surface motion using a laser-based respiratory gating system, and respiratory waveform adjustments were manually performed by the technologist. In contrast, in the VF workflow, the technologist initially adjusted the respiratory signal based on the laser output and subsequently provided the patient with VF. After this initial adjustment, patients actively regulated their own breathing by observing the displayed waveform, while technologists continued to monitor the signal and intervened if necessary.

### Effect of visual feedback on treatment time reduction

Treatment time was defined as the total beam delivery time, calculated as the sum of the irradiation durations for the first and second treatment fields, from beam-on to beam-off for each field. This definition therefore includes the beam-on time, waiting time for the respiratory signal to enter the gating window, and any beam-related delays occurring during gated delivery, while patient setup and imaging times were excluded.

The average treatment time of each patient over the 4 treatments was compared in each VF patient and the corresponding non-VF patients, and the effect of the reduced treatment time for VF was calculated. The difference was calculated using Eq. 1:

(1) $$\begin{array}{c} \mathrm{Difference}[\%]=\frac{{\mathrm{Treatment}\;\mathrm{time}}_{\mathrm{VF}}-{\mathrm{Treatment}\;\mathrm{time}}_{\mathrm{non}-\mathrm{VF}}}{{\mathrm{Treatment}\;\mathrm{time}}_{\mathrm{non}-\mathrm{VF}}}\times 100 \end{array}$$

${\mathrm{Treatment}\;\mathrm{time}}_{\mathrm{VF}}$ represents the average treatment time over 4 sessions in a VF patient, and ${\mathrm{Treatment}\;\mathrm{time}}_{\mathrm{non}-\mathrm{VF}}$ denotes the average treatment time over 4 sessions in non-VF reference patients.

To directly reflect beam delivery efficiency under respiratory-gated irradiation, the duty cycle (DC) was used. The DC was defined as the ratio of the beam-on time to the total treatment time and was expressed as a percentage, as shown in the following Eq. 2:

(2) $$\begin{array}{c} \mathrm{DC}[\%]=\frac{\mathrm{Beam}-\mathrm{on}\;\mathrm{time}}{\mathrm{Treatment}\;\mathrm{time}}\times 100 \end{array}$$

Beam-on time was defined as the cumulative duration during which the therapeutic beam was actively delivered to the patient, explicitly excluding beam-off intervals due to respiratory gating or manual interruption. Treatmenttime includes beam-off intervals resulting from respiratory gating or manual beam interruption.

### Respiratory waveform reproducibility

To quantify respiratory variability, the root mean square error (RMSE) and standard deviation were calculated.^36^ The RMSE value was calculated using Eq. 3:

(3) $$\begin{array}{c} \mathrm{RMSE}(i)=\sqrt{\frac{\sum^{N}{\left(x_{i}{-\overset{®}{x}}_{i}\right)}^{2}}{N}} \end{array}$$

*x*_*i*_ represents the measured waveform position (mm) at phase i, ${\overset{®}{x}}_{i}$ is the mean waveform position (mm) at phase i, and N is the total number of analyzed waveforms. Waveform data were collected for 1 minute from the start of the first field irradiation and 1 minute after the end of the first field irradiation. The reason for collecting respiratory waveform data during the first and last 2 minutes of the first field was to capture time-dependent irregular changes in the respiratory pattern.^37^ The same process was repeated for the second field, resulting in a total of 4 minutes of respiratory waveform data. Because treatment time varies depending on tumor size and differences in treatment planning, the total duration of respiratory waveform acquisition would differ among patients if the entire treatment session were analyzed. To avoid imbalance in the amount of waveform data between patients, a fixed sampling period was predefined. This time-based sampling approach allowed standardization of the data volume and enabled a more balanced comparison of respiratory reproducibility. To compare RMSE values across different phases, RMSE values were calculated from the waveform data at phases 25%, 50%, 75%, and 100% over the 4 minutes recording period. To evaluate the variability of the entire waveform, the average RMSE value for each phase was calculated to obtain the overall RMSE.

Overall and phase-specific RMSE values were compared between VF and non-VF patients to assess the effect of reducing respiratory variability in VF. The difference was calculated using the following Eq. 4:

(4) $$\begin{array}{c} \mathrm{Difference}[\%]=\frac{{\mathrm{RMSE}}_{\mathrm{VF}}-{\mathrm{RMSE}}_{\mathrm{non}-\mathrm{VF}}}{{\mathrm{RMSE}}_{\mathrm{non}-\mathrm{VF}}}\times 100 \end{array}$$

${\mathrm{RMSE}}_{\mathrm{VF}}$ represents the RMSE value for VF patients, and ${\mathrm{RMSE}}_{\mathrm{non}-\mathrm{VF}}$ denotes the RMSE value for non-VF patients.

### Statistical analysis

To assess the statistically significant differences between VF and non-VF patients, data were initially evaluated for normality and homogeneity of variance. The paired *t*-test was used using Microsoft Excel. A *P* value of <.05 indicated statistically significant differences. The 95% confidence interval (95% CI) was calculated to estimate the magnitude and precision of the effect.

Correlation analysis was performed to evaluate the relationships between the differences in DC and RMSE and the corresponding reduction in treatment time for each matched pair. Pearson’s correlation coefficient was used to quantify the strength of these associations.

## Results

### Characteristics of the patient

The respiratory waveforms and treatment times of 10 VF patients and 23 historical non-VF patients were analyzed. Table 1 presents the characteristics of the patients who were included in this study. The amplitude was calculated as a percentage of the peak-to-peak amplitude of the respiratory waveform for each patient.

**Table 1:** Combination of non-VF patient.

Patients	VF		Non-VF	
	CTV [cc]	Amplitude [%]	CTV [cc]	Amplitude [%]
Pair1	103.88	12	100.42/108.77/91.61	9/11/15
Pair2	157.68	12	148.98/156.21	14/10
Pair3	190.57	14	182.94 /211.25	19/11
Pair4	123.85	14	112.66/122.26	13/12
Pair5	120.33	16	113.11/112.66/125.21	18/13/17
Pair6	183.04	22	192.28/182.94/184.27	23/19/25
Pair7	327.1	24	327.65	29
Pair8	62.69	19	49.54/57.64	12/18
Pair9	246.03	24	259.43/264.77/256.64	22/2/28
Pair10	1106.24	20	944.96/1182.12	15/15

The non-VF patient selected from past cases who resembled VF patient.

Most patients were more likely to have tumor volumes ranging from approximately 100 to 300 cc. Pair 10, which had the largest tumor size, exhibited volumes between approximately 945 and 1182 cc.

### Treatment time

Table 2 shows the treatment times for VF patients and the average treatment time for non-VF patients. The treatment time was reduced in 8 of 10 pairs. Further, in 6 of 8 pairs, the treatment time was reduced by >5 minutes when using VF compared with non-VF. The mean difference showed a reduction of 27.9%. Pair 10 had the largest reduction in treatment time, with a difference of 45.8%. Conversely, in pair 3, the VF patient required a longer treatment time, with a difference of approximately 1 minute, 29 seconds. Treatment time was significantly shorter in the VF group, with a mean paired difference of 4.90 minutes (95% CI, 1.55-8.25 minutes; *P* < .05; paired *t*-test).

**Table 2:** Treatment time of non-VF patients and a VF patient.

Patients	Irradiation time[min]		Difference	*p* value
	VF	Non-VF	[％]	
Pair1	9:03	14:12 ± 2:53	−36.3	
Pair2	17:30	19:53 ± 0:59	−12.0	
Pair3	18:36	17:07 ± 0:39	8.7	
Pair4	8:41	14:09 ± 2:48	−39.6	
Pair5	13:14	20:36 ± 2:54	−36.1	
Pair6	9:36	16:04 ± 2:13	−40.2	
Pair7	10:55	13:16 ± 0:00	−18.3	
Pair8	10:37	10:22 ± 2:23	2.4	
Pair9	9:49	15:32 ± 4:24	−36.8	
Pair10	18:41	34:27 ± 10:41	−45.8	
Average	12:40	17:34 ± 2:59	−27.9	0.010*

The results confirm that the use of VF shortened the treatment time in 8 out of 10 pairs.

According to Table 3, the DC was highest in pair 10, with a value of 15.21%. The difference in DC between VF and non-VF was also largest for pair 10, reaching 87.5%. Overall, the mean DC was higher in the VF group than in the non-VF group. However, in pairs 3 and 8, the DC was higher in the non-VF condition than in the VF condition.

**Table 3:** DC of non-VF patients and a VF patient.

Patient	Duty cycle[%]		Difference [％]	*p* value
	VF	Non-VF		
Pair1	8.78	6.12 ± 3.10	47.1	
Pair2	8.84	7.47 ± 2.06	20.4	
Pair3	6.50	7.03 ± 0.09	−7.6	
Pair4	7.00	4.36 ± 0.11	60.5	
Pair5	8.74	6.44 ± 1.02	39.7	
Pair6	9.85	6.00 ± 0.07	66.6	
Pair7	11.17	8.74 ± 0.00	25.9	
Pair8	9.86	10.42 ± 0.51	−4.1	
Pair9	12.02	8.57 ± 3.55	40.1	
Pair10	15.21	8.47 ± 1.01	87.5	
Average	9.80	7.36 ± 1.15	37.61	0.006*

An increased DC with VF was observed in 8 of the 10 patients.

### Respiratory waveform reproducibility

**Figure 2:**
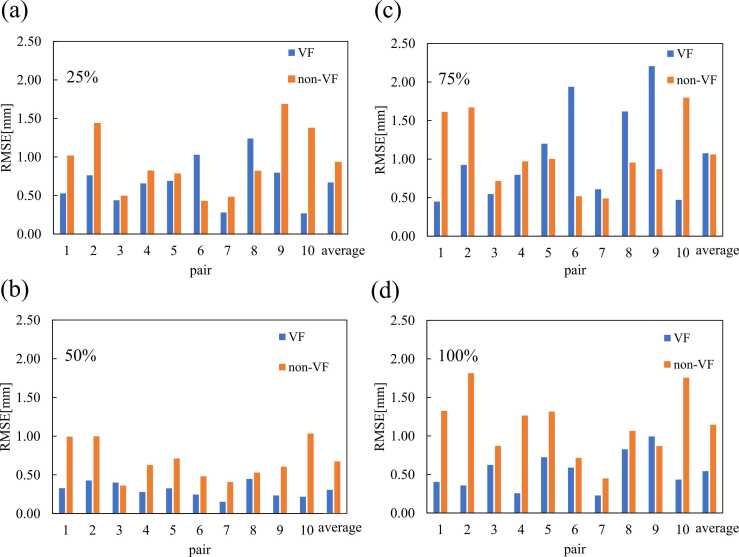
(a) The RMSE of non-VF and VF differs at phase 25%, (b) at phase 50%, (c) at phase 75%, and (d) at phase 100%. Furthermore, the use of VF led to a reduction in RMSE for many pairs at phases 50% and 100% (*P* < .01).

Table 4 and Figure 2 present the RMSE value at each phase. The RMSE value was significantly reduced at phases 50 (95% CI, 0.19-0.55 mm; *P* < .01; paired *t*-test) and 100% (95% CI, 0.23-0.97 mm; *P* < .01; paired *t*-test). The RMSE value decreased by 28.2% in the VF group compared with the non-VF group (based on Table 4). However, the value was not significant at phase 25% (95% CI, −0.12 to 0.65 mm; *P* = .079; paired *t*-test). At phase 75%, the RMSE values for non-VF and VF were almost similar, with a difference corresponding to a 1.4% increase.

**Table 4:** The RMSE at each respiratory phase for individual patients pair is shown.

Phase	RMSE[mm]		Difference [%]	*p* value
	VF	Non-VF		
25%	0.67 ± 0.29	0.93 ± 0.48	−28.2	0.158
50%	0.31 ± 0.11	0.67 ± 0.33	−54.7	0.002*
75%	1.08 ± 0.61	1.06 ± 0.55	1.4	0.959
100%	0.54 ± 0.24	1.14 ± 0.42	−52.5	0.007*

The use of the VF system resulted in a general reduction in RMSE across all phases except at phase 75%.

The overall RMSE value in the VF group was reduced by 32.0% compared with that in the non-VF group. Although the difference was not statistically significant, VF was effective in improving the respiratory waveform reproducibility in most pairs (95% CI, −0.05 to 0.66 mm; *P* = .086, paired *t*-test). However, respiratory reproducibility did not improve in all cases. The greatest reduction in RMSE was observed in pair 10, with a difference of −76.7%. The smallest reduction effect was observed in pair 6, where the RMSE value increased by 77.7%.

### Relationship between root mean square error, duty cycle, and treatment time

Correlation analysis using the VF-non-VF differences demonstrated a significant negative correlation between DC and treatment time (*r* = −0.72, *P* = .018). In contrast, no significant correlation was observed between RMSE and DC (*r* = −0.30, *P* = .40). A moderate positive correlation was observed between RMSE and treatment time, although this did not reach statistical significance (*r* = 0.56, *P* = .09).

## Discussion

### Summary of the study

This study evaluated the effects of VF on respiratory waveform reproducibility and reduction in treatment time in CIRT. To the best of our knowledge, this is the first prospective study to specifically investigate the treatment time–reduction effect of VF in respiratory-gated CIRT. The primary clinical significance of this study lies not merely in confirming improved respiratory reproducibility with VF but in demonstrating its potential to enhance treatment efficiency specifically in synchrotron-based CIRT, where treatment duration is particularly susceptible to respiratory instability.

### Treatment time

As shown in Table 2, the mean treatment time was significantly reduced in VF patients compared with non-VF patients, thereby suggesting improved time efficiency in irradiation sessions. Linthout et al reported that the treatment time was reduced by 17.6% with the introduction of VF in X-ray therapy.^17^ In comparison, this study observed a 28% reduction in treatment time, which indicated that the impact of VF on treatment time can be greater in CIRT. However, it should be noted that VF and non-VF were compared between different but clinically similar patients rather than within the same individuals. In addition, the small sample size and the use of matched historical controls limit causal inference regarding the magnitude of the observed time reduction. Because respiratory patterns differed between the 2 conditions, treatment plans derived from 4DCT also differed; therefore, the observed reduction in treatment time reflects not only the effect of VF itself but also condition-specific treatment planning and should be interpreted with caution. VF improved the reproducibility of respiratory waveforms, which likely increased the number of usable irradiation periods. Unlike Linthout et al., the present study did not incorporate training sessions, which may help patients gain a deeper understanding of the VF system and further improve respiratory reproducibility.^38^, ^39^

Previous studies on respiratory-gated irradiation have demonstrated the importance of analyzing the dependence of treatment delivery on DC.^18^ In this study, DC values were higher in the VF group than in the non-VF group. VF and non-VF were compared, pairs with larger differences in DC tended to exhibit shorter treatment times, suggesting that the VF system was associated with more efficient beam delivery (95% CI, 0.84%-4.04%; *P* < .01; paired *t*-test).

No significant differences were observed in CTV size (95% CI: −13.35 to 20.36 cc, *P* = 0.67) or gating amplitude (95% CI: −1.04 to 2.60%, *P* = 0.357) between the VF and non-VF groups. These findings suggest that the difference in treatment time associated with VF application was not attributable to differences in CTV size or gating amplitude between the 2 groups.

Synchrotron-based CIRT is characterized by inherently intermittent beam extraction, resulting in more restricted effective irradiation windows compared with quasi-continuous beam delivery in photon therapy. Effective dose delivery requires temporal coincidence between beam availability and the predefined respiratory gating window. Consequently, respiratory irregularities not only reduce the DC but also increase the likelihood of temporal mismatches between beam spill and the gating window, thereby decreasing overall beam utilization efficiency. Under non-VF conditions, these combined effects may lead to relatively prolonged treatment times compared with photon therapy. Stabilization of respiratory motion by VF may improve gating regularity and increase the probability of beam-gate synchronization, potentially mitigating these inefficiencies.

### Respiratory waveform reproducibility

The improvement in RMSE could be a potential factor contributing to a reduced treatment time in this study. As shown in Table 4, Table 5, the RMSE values of the VF patients were lower than those of the reference non-VF patients. Thus, VF improved the respiratory waveform reproducibility. This result is consistent with that of the study conducted by Nakajima et al.^16^ Several studies have shown that VF or audiovisual feedback improves respiratory reproducibility and time efficiency in irradiation sessions. George et al quantified respiratory variability in 24 patients with lung cancer using standard deviation and reported improved gating efficiency and reproducibility with audio VF (AVF).^18^ Other studies have also found similar effects.^20^, ^23^, ^24^ These findings are consistent with the results of the present study and align with previous reports indicating that VF can improve respiratory signal reproducibility. The RMSE value was calculated at each phase (25%, 50%, 75%, and 100%), thereby revealing a significant reduction in the RMSE value at phases 50% and 100% (*P* < .01). This suggests that VF enhances the visibility of the upper and lower limits of respiration and contributes to improved respiratory reproducibility. At phases 25% and 75%, which correspond to the midrange of the breathing cycle, no significant RMSE reduction was observed, likely due to the lack of clear visual markers in these phases.

**Table 5:** Overall RMSE at each phase.

Patients	Overall RMSE [mm]		Difference [%]	*p* value
	VF	Non-VF		
Pair1	0.43 ± 0.16	1.70 ± 0.85	−75.0	
		0.59 ± 0.74	−27.6	
		1.42 ± 0.49	−69.9	
Pair2	0.62 ± 0.27	1.09 ± 0.69	−43.5	
		1.87 ± 0.67	−66.9	
Pair3	0.50 ± 0.18	0.73 ± 0.59	−31.3	
		0.49 ± 0.35	2.5	
Pair4	0.50 ± 0.26	0.69 ± 0.52	−27.9	
		1.15 ± 0.50	−56.8	
Pair5	0.73 ± 0.47	0.49 ± 0.63	49.2	
		1.21 ± 0.66	−39.4	
		1.15 ± 0.50	−36.2	
Pair6	0.95 ± 0.63	0.34 ± 0.25	181.7	
		0.73 ± 0.50	29.8	
		0.53 ± 0.55	80.2	
Pair7	0.32 ± 0.19	0.46 ± 0.51	−30.5	
Pair8	1.03 ± 0.45	0.81 ± 0.57	27.4	
		0.87 ± 0.33	18.5	
Pair9	1.06 ± 0.43	1.23 ± 0.93	−14.2	
		0.52 ± 0.46	103.8	
		0.73 ± 0.95	45.7	
Pair10	0.35 ± 0.16	1.61 ± 0.63	−78.4	
		1.37 ± 0.57	−74.7	
Average	0.65 ± 0.32	0.95 ± 0.60	−32.0	0.086

VF reduced RMSE in many patient pairs.

In 2 of 10 cases, the treatment time did not decrease with VF compared with the reference non-VF. The difference in RMSE between VF and non-VF at phase 50% was smaller in pairs 3 and 8, where the treatment time increased with the use of VF (Table 2). This indicates that the improved RMSE in phase 50% may contribute to the shorter treatment time.

These findings may also be applicable to the treatment of other tumors in respiratory-moving organs, such as lung and pancreatic cancers, which suggests promising implications beyond liver cancer.

This study used external respiratory signals. Clinically, respiratory gating relies on the correlation between external respiratory signals and tumor motion (internal respiratory signals). Several studies have reported a correlation between the external and internal respiratory signals.^40^, ^41^, ^42^ Based on these studies, improving the reproducibility of external respiratory signals can contribute to enhanced treatment accuracy. However, the correlation between the external and internal respiratory signals is not always strong.^8^, ^43^, ^44^

Nevertheless, previous studies have shown that the use of AVF can strengthen the correlation between external and internal respiratory signals.^45^, ^46^

Goossens et al found that in all 13 patients with nonsmall-cell lung cancer, a high internal/external correlation reproducibility was achieved for tumor motion in the vertical direction, and the correlation reproducibility in the anteroposterior direction improved with AVF.^46^ In the present study, audio instructions were not incorporated, and correlations in abdominal tumors such as liver cancer were not evaluated, indicating the need for further research.

A limitation of this study is that VF and non-VF were compared using different but similar patients, rather than within the same individual. VF and non-VF involve different respiratory patterns. Hence, the treatment plans created from the tumor respiratory motion obtained via 4DCT scan differ between the 2 conditions. In addition, conducting the study within the same patient would require rescanning with CT scan and replanning, which poses an increased risk of radiation exposure. Due to these reasons, the current study was not conducted within the same patients. Therefore, the individual effects of VF on respiratory waveform reproducibility and treatment time reduction cannot be completely identified. In addition, the correlation between external respiratory surrogates and internal tumor motion was not directly assessed in this study. Therefore, the effect of VF on internal tumor motion cannot be fully determined. Moreover, this study only included 10 VF patients. Respiratory waveform data were collected from patients with CTVs ranging from 49.54 to 1182.12 cc. However, none of the patients presented with GTVs between 400 and 900 cc. By increasing the sample size, a more detailed analysis, including the correlation between GTV and reduction in treatment time, can be performed. Furthermore, this study was not randomized and represents a between-patient comparison. Because the number of liver CIRT cases at our institution was limited during the study period, only a small number of clinically comparable non-VF patients were available for some VF patients, making random selection difficult. Therefore, the possibility of selection bias cannot be excluded. Future studies with a larger patient cohort and a prospective randomized design are warranted to further validate these findings.

### Relationship between root mean square error, duty cycle, and treatment time

Correlation analysis using the VF-non-VF differences was performed to investigate the relationships among RMSE, DC, and treatment time. A significant negative correlation was observed between DC and treatment time (*r* = −0.72, *P* = .018), indicating that greater increases in DC were associated with larger reductions in treatment time. No significant correlations were observed between RMSE and DC (*r* = −0.30, *P* = .40). Although not statistically significant, a moderate positive correlation was observed between RMSE and treatment time (*r* = 0.56, *P* = .09), suggesting that larger improvements in respiratory reproducibility may be associated with greater reductions in treatment time. This relationship may reach statistical significance with a larger sample size. In addition, the mean respiratory cycle duration was significantly longer in the VF group than in the non-VF group (4.97 ± 1.20 seconds vs 3.83 ± 0.53 seconds; 95% CI, 0.08-2.19 seconds; *P* < .05; paired *t*-test). A relatively longer respiratory cycle may slightly facilitate beam-gate synchronization by extending the duration of each gating phase, potentially improving beam utilization. However, no significant correlations were observed between respiratory cycle duration and either treatment time (*r* = 0.32, *P* = 0.37) or DC (*r* = −0.14, *P* = .70), and these associations were weaker than those observed for RMSE. These findings suggest that the contribution of respiratory cycle prolongation to treatment efficiency is limited.

## Conclusion

This study evaluated the effects of VF on respiratory reproducibility and time efficiency in irradiation sessions in respiratory-gated CIRT. The results suggest that VF improves respiratory waveform reproducibility, which may support improved treatment accuracy. Furthermore, the greater reduction in treatment time compared to respiratory-gated X-ray therapy indicates the potential for improved time efficiency in irradiation sessions.

## CRediT authorship contribution statement

Ren Umetani - writing the manuscript and data analysis; Yuya Miyasaka - creating treatment plan and data analysis and review manuscript; Hikaru Souda - review data and review manuscript; Hongbo Chai - review data and review manuscript; Miyu Ishizawa - review data and review manuscript; Yasuhito Hagiwara - contouring ROIs, clinical integration, clinical review and review manuscript; Takashi Kaneko - contouring ROIs, clinical integration, clinical review and review manuscript; Yoshifumi Yamazawa - review data and manuscript review; Hiraku Sato - clinical integration, clinical review and review manuscript; Masashi Koto - review data and manuscript review; Takeo Iwai - management and coordination responsibility for the research activity planning and execution.

## Declaration of Conflicts of Interest

The authors declare that they have no known competing financial interests or personal relationships that could have appeared to influence the work reported in this paper.

## Data Availability

Research data are stored in an institutional repository and will be shared upon request to the corresponding author.
